# Eye Cups

**Published:** 1872-04

**Authors:** 


### "EYE CUPS."

We had supposed that the "eye cup" swindle had long since been exploded, and that no sensible person could be found at this day, silly enough to waste money upon such a device.—But, it seems that notwithstanding the cautions that have been published through this, and other publications, against their use, the business of manufacturing and selling this dangerous implement has taken a new impetus, and upon a scale that is likely to attract the attention of many people, greatly to their injury.

Years ago, in 1851 we think it was, Mr. J. Ball, of this city, conceived the idea of constructing an apparatus that, by elongating the axis of the eye, would increase the power of its lenses, and so enable a long sighted eye to see without aid of spectacles. He took his cue from the published experiments of John Quincy Adams, who, it will be remembered, retained normal sight,—being able to read without glasses—to the day of his death, and which blessing he attributed to rubbing or pressing his eyes, with thumb and finger, in their horizontal axis, several times daily. His explanation was, that by this procedure his eye was elongated, and so prevented from becoming "old sighted." Acting upon this theory, Mr. Ball constructed his eye cups, which were widely known, and extensively sold, under the name of "Dr. J. Ball's Eye Restorers." Many persons of intelligence gave their approval to this instrument, which doubtless, immediately after use, afforded better vision; but the moment the humors were allowed to regain their normal position, the eye became long sighted, as before. Some persons were satisfied to take up their eye cups, apply them to their eyes for a minute, and then pick up their paper and read until the elongation had subsided, when they were reapplied. In this manner they used them month after month, or, until severe congestion of the eye ensued and vision was seriously injured or completely destroyed, when they abandoned further use of them. The deleterious and dangerous effects of the instrument becoming known, the manufacture and sale of the eye-cup was abandoned for a while but was soon resurrected under what purported to be an improvement upon the old device, and which was again extensively advertised as "Cornea Restorers," "Eye Sharpeners," "Old Sight made new," etc. But so much injury resulted from their use that the traffic again fell into dis repute, and this time, we fondly hoped, forever. We were therefore much amazed upon picking up *Harper's Weekly*, recently, and reading the following conspicuous advertisement:

**SAVE YOUR EYES,**

Throw away your spectacles! Avoid a surgical operation by reading our illustrated "PHYSIOLOGY OF THE EYE AND SIGHT" and Near-Sightedness. It treats on Impaired Vision, Weak, Watery, Sore and Inflamed Eyes, and the worst disorders of the Eye. Mailed free to any address by

**New York College of Health,**

Box 840 P. O. 165 & 167 Broadway, N. Y.

This, we at once concluded, must be another form of the cup swindle; therefore, through the agency of a friend, ordered this wonderful work, which promised so much for suffering humanity. We were not disappointed, for when we had the "Physiology of the Eye and Sight," in our possession, we found it to be a pamphlet written by some ignoramus in the interest of the same old eye-cup. But this machine is said to be wonderfully "improved," and the pamphlet declares all other forms of the cup to be worthless and dangerous. The present one, however, is the "PATENT DUPLEX EYE-SIGHT RESTORER OR EYE BOWLES," a double-barreled sort of thing, warranted we suppose, to kill in half the time required by the single-barreled sort.

We find upon examining the pamphlet that *this* eye cup is placed upon the market by the "New York College of Health," which fact, of course, gives the thing character at once. No one would suspect that a *College* would ever place a worthless, much less a dangerous article in the hands of the public, and then, the Duplex fellow is so thoroughly fortified behind page after page of certificates of cure, rendering his argument perfectly irresistible.

Would you believe it? this patent-duplex-double-barreled-contrivance, will cure *any* disease of the eye, or any sort of blindness, ever invented by a reckless people! It is "good for," and will certainly *cure*, amblyopia, presbyopia, myopia, asthenopia, epiphora, opthalmia, amurosis, photophobia, ptosis, sore eyes, "hygenic messengers"— no, we are mistaken about the last mentioned disease, that's a *medicine*, which the College sells with the double-barreled arrangement. It isn't sure fire unless loaded with the "hygenic messenger," so please remember, for we have the authority of the pamphlet for saying that they cannot "promise a permanent *cure of sight*," without the afore mentioned messenger. In perusing this precious document we also learn that the price of the duplex thing is $9.50; but, in order to convince you that they, the "New York College of Health," "desire to do the greatest good to mankind—to restore sight to any one willing to ask for it--and to convince the skeptical that they do not care for their money," why, they will send any one the double-barreled-duplex-eye-restorer, for $6.-50; giving them the privilege of sending the balance, of four dollars, when they are satisfied that it has done them good. Now that's clever! Who could resist such an offer? Of course, hundreds of innocents avail themselves of this opportunity of getting the Duplexer for four dollars less than cost, by being just a little skeptical. If any of our readers are so unfortunate as to be among the number, we will inform them just what they may expect from the use of this, or any other eye cup:

In the first place, presbyopia, or "old sightedness," as it is familiarly called, does *not* depend solely upon the flattening of the cornea or the lens, as is claimed by these eye-cup charlatans. The defect in the vision of aged people, is caused by a loss of accommodation of the eye, the result of inactivity of the ciliary muscle and suspensory ligament of the lens, as well as the loss of elasticity in the lens itself, from which it loses its power of assuming a more convex form, necessary for close adjustment, when the tension of the suspensory ligament is relaxed. By reason of these defects, the aged person is unable to accommodate his eye to near objects; and all the eye cups in christendom, duplex and double-barreled, cannot restore this lost function. Then, the theory that the eye can have its form altered by continued pressure, or by rubbing it in one direction, is erroneous. The eye being an elastic globe, filled with fluid and gelatinous substances, *cannot* be pressed or manipulated into any but its natural shape, any more than you could cause the rubber ball of the eye-cup to retain its flatness by pressing upon it with your fingers. The moment you remove pressure from the ball, that instant the cup regains its former shape,—so does the eye.

Not only is the cup of no avail in permanently changing the shape of the eye, but is absolutely dangerous, when its use is persisted in. We have known the lens to be dislodged, in three instances, by its use. Have seen a rupture of a small retinal artery, and separation of the retina, by its application. All of these cases resulted in blindness. Every ophthalmic surgeon can give instances of very serious injury, and often blindness, produced by the application of eye-cups. To think for a moment, that *diseases* of the eye can be remedied by their use, is too absurd. Any one who imagines that inflammation of an eye can be subdued by attracting more blood to it, by means of "suction," or atmospheric pressure, must be a ninny, indeed; ophthalmic surgeons usually endeavor to abstract blood by leeching and otherwise, from the region of the eye, when in a high state of inflammation. There are very many forms of disease of the eye wherein the application of the eye-cup would be fatal; in that form of partial blindness caused by the separation of the retina, for instance, the retina would be still more separated by action of the cup. In ulceration of the cornea, the application of "suction" would at once cause the globe of the eye to discharge its contents. In short, we know of no form of impaired vision where the application of atmospheric pressure would be of the slightest permanent benefit; but we do know of an infinite variety of diseases of the eye where its application would be fatal to vision. Therefore, the deductions to be drawn from our conclusions are, let all manner of eye cups alone, and trust not your precious vision to the manipulations of advertising charlatans.

